# Lanthanide-Doped ZnO Nanoparticles: Unraveling Their Role in Cytotoxicity, Antioxidant Capacity, and Nanotoxicology

**DOI:** 10.3390/antiox13020213

**Published:** 2024-02-08

**Authors:** Jorge L. Mejía-Méndez, Diego E. Navarro-López, Araceli Sanchez-Martinez, Oscar Ceballos-Sanchez, Luis Eduardo Garcia-Amezquita, Naveen Tiwari, Karla Juarez-Moreno, Gildardo Sanchez-Ante, Edgar R. López-Mena

**Affiliations:** 1 Laboratory of Phytochemistry Research, Chemical Biological Sciences Department, Universidad de las Américas Puebla, Ex Hacienda Sta. Catarina Mártir S/N, San Andrés Cholula 72810, Mexico; jorge.mejiamz@udlap.mx; 2 Tecnologicode Monterrey, Escuela de Ingeniería y Ciencias, Av. Gral. Ramón Corona No 2514, Colonia Nuevo México, Zapopan 45121, Mexico; diegonl@tec.mx; 3 Departamento de Ingeniería de Proyectos, Centro Universitario de Ciencias Exactas e Ingenierías (CUCEI), Universidad de Guadalajara, Av. José Guadalupe Zuno # 48, Industrial Los Belenes, Zapopan 45157, Mexico; araceli.sanchez46@academicos.udg.mx (A.S.-M.); oscar.ceballos@academicos.udg.mx (O.C.-S.); 4 Tecnologico de Monterrey, Escuela de Ingeniería y Ciencias, Av. Eugenio Garza Sada No 2501, Monterrey 64849, Mexico; garcia.amezquita@tec.mx; 5 Center for Research in Biological Chemistry and Molecular Materials (CiQUS), University of Santiago de Compostela, Rúa Jenaro de La Fuente S/N, 15782 Santiago de Compostela, Spain; 6 Centro de Física Aplicada y Tecnología Avanzada (CFATA), Universidad Nacional Autónoma de México (UNAM), Querétaro 76230, Mexico

**Keywords:** lanthanide elements, antioxidant activity, in vivo toxicity, machine learning modeling, sonochemical synthesis

## Abstract

This study used a sonochemical synthesis method to prepare (La, Sm)-doped ZnO nanoparticles (NPs). The effect of incorporating these lanthanide elements on the structural, optical, and morphological properties of ZnO-NPs was analyzed. The cytotoxicity and the reactive oxygen species (ROS) generation capacity of ZnO-NPs were evaluated against breast (MCF7) and colon (HT29) cancer cell lines. Their antioxidant activity was analyzed using a DPPH assay, and their toxicity towards *Artemia salina* nauplii was also evaluated. The results revealed that treatment with NPs resulted in the death of 10.559–42.546% and 18.230–38.643% of MCF7 and HT29 cells, respectively. This effect was attributed to the ability of NPs to downregulate ROS formation within the two cell lines in a dose-dependent manner. In the DPPH assay, treatment with (La, Sm)-doped ZnO-NPs inhibited the generation of free radicals at IC_50_ values ranging from 3.898 to 126.948 μg/mL. Against *A. salina* nauplii, the synthesized NPs did not cause death nor induce morphological changes at the tested concentrations. A series of machine learning (ML) models were used to predict the biological performance of (La, Sm)-doped ZnO-NPs. Among the designed ML models, the gradient boosting model resulted in the greatest mean absolute error (MAE) (MAE 9.027, R^2^ = 0.86). The data generated in this work provide innovative insights into the influence of La and Sm on the structural arrangement and chemical features of ZnO-NPs, together with their cytotoxicity, antioxidant activity, and in vivo toxicity.

## 1. Introduction

Antioxidants are a class of natural and synthetic molecules that can donate electrons to free radicals rampaging through the body, neutralizing them and diminishing their ability to cause damage [1]. Over the last few decades, natural antioxidants such as carotenoids, flavonoids, and polyphenols, due to their multiple functional groups, have been used to restore the oxidative stress balance while exerting additional biological activities that have anti-aging, anti-inflammatory, and anti-cancer effects [2]. However, the application of natural antioxidants is often limited by their susceptibility to degradation and high sensitivity to temperature, pH, and light [3,4]. In contrast to natural antioxidants, synthetic antioxidants are an attractive alternative due to their high antioxidant activity, stability, and reproducibility [5].

Nanotechnology is an emerging research field where objects on the nanometric scale are manipulated for agricultural, industrial, electronic, and medical applications [6]. Used as antioxidants, nanomaterials can disrupt the generation of free radicals through various mechanisms due to their capacity to mimic the activity of enzymes or to donate electrons [7]. These phenomena can occur through two main mechanisms: prevention and chain breaking. In the prevention mechanism, nanomaterials can limit the generation of free radicals via indirect interaction [8]. Typical examples where this indirect interaction is observed include during the chelation of transition metals, photo-oxidation processes, and oxygen scavenging [9]. In the chain-breaking mechanism, nanomaterials downregulate the propagation reaction by interacting with free radicals faster than the oxidizable substrate [10]. Given the importance of synthetic antioxidants in biomedicine, their evaluation in vitro and in vivo has become an active research field in recent decades. The in vitro and in vivo performance of several nanomaterials, for example, nanopowders, nanoflowers, nanowires, nanocrystals, and metal-oxide and rare earth nanoparticles (NPs), have been reviewed so far [11].

In human healthcare, rare earth (RE) nanoparticles are used for drug delivery, tumor therapy, bioimaging, and diagnosis [12]. The development of these types of nanomaterials can include the use of essential trace elements such as zinc (Zn^2+^), which is a safe material exploited for nanobiotechnological uses due to its optical, mechanical, catalytic, and intrinsic therapeutic properties and its ease of manipulation in the synthesis of nanostructures doped with lanthanides [13,14]. In therapeutic applications, lanthanide-doped nanoparticles have been reported to target neurological diseases and possess biocompatibility with treatment regimens such as chemotherapy and radiotherapy [15,16]. In comparison to other nanostructures derived from lanthanide elements, lanthanum oxide (La_2_O_3_) is utilized to fabricate nanostructures through a variety of physical and chemical methods and is recognized for its important biomedical uses due to its low genotoxicity, the presence of 4*f* shielded electrons, its biocompatibility, and its intrinsic fluorescent and magnetic features and redox-switching capacity [17]. In the same context, Sm_2_O_3_ constitutes an attractive rare earth element for developing or doping materials with nanometric architectures and therapeutic applications due to its five 4*f* electrons, enhanced absorbance of infrared radiation, chemical stability, and catalytic activity [18]. However, the biological performance of ZnO-NPs co-doped with La^3+^ and Sm^3+^ has not been studied.

In recent years, machine learning has become a powerful tool for predicting the useful properties of materials and providing insights into the different mechanisms behind the interaction of the material with the environment [19,20]. An initial dataset is required to train machine learning models. Such a dataset is constructed by gathering information from experiments [21]. The dataset incorporates the measurements of several independent variables that describe material characteristics such as structural, optical, and morphological traits, among others, and one response to be modeled. Once the model is constructed from those data, it can be used in different ways, such as to predict values or to understand what the relevance of each variable is.

Herein, (La, Sm)-doped ZnO NPs were synthesized using a polymerized solution method and modified using an ultrasonic generator probe. The effect of incorporating La^3+^ and Sm^3+^ was analyzed through their structural, optical, and morphological properties. The cytotoxicity of the prepared NPs was tested against breast and colon cancer cell lines. To continue exploring their biological activities, their capacity to induce or decrease the generation of reactive oxygen species (ROS) was studied in both cell lines. In addition, their antioxidant activity was investigated using the DPPH assay, and their toxicity was evaluated in *Artemia salina* nauplii as an in vivo model. Machine learning algorithms were used to predict antioxidant activity, providing new insight into the role of doping in cytotoxicity, antioxidant activity, and nanotoxicology.

## 2. Materials and Methods

### 2.1. Nanomaterials Synthesis

The RE-doped nanomaterials were prepared using the polymerized solution method reported in [22]. However, an adjustment was made during the stirring process. The solutions were exposed to ultrasonic treatment for 15 min. The ultrasonic generator probe was directly submerged in the solutions, in a cycle of 40 s on and 20 s off at an amplitude of 70% (Qsonica Q700 sonicator, 20 kHz). The chemicals used were polyvinyl alcohol (PVA, a.m.w.: 70,000–1000, 87–90% hydrolyzed, Sigma-Aldrich, St. Louis, MO, USA), sucrose (C_12_H_22_O_11_, ACS reagent, Sigma-Aldrich), citric acid (C_6_H_8_O_7_H_2_O, ACS reagent, Sigma-Aldrich, St. Louis, MO, USA), Zn(NO_3_)_26_H_2_O (98%, Sigma-Aldrich, St. Louis, MO, USA), La(NO_3_)_36_H_2_O (99%, Sigma-Aldrich, St. Louis, MO, USA), and Sm(NO_3_)_36_H_2_O. The developed nanomaterials were labeled Z, ZL, ZLS, and ZS for undoped, La-, (La, Sm)-, and Sm-doped ZnO.

### 2.2. Characterization of Nanomaterials

The effect of incorporating La^3+^ and Sm^3+^ in the crystal structure of ZnO nanoparticles was characterized using X-ray diffraction (XRD) with a Cu anode, λ = 1.5406 Å (Empyrean, PANalytical, Westborough, MA, USA). XRD patterns were obtained from 20° to 75° (2θ) with a 0.01° step size. Attenuated total reflectance Fourier-transform infrared ATR-FTIR (Shimadzu, IRAffinity, Columbia, MD, USA) was used to verify the presence of organic compounds. The spectra were recorded in the 4000–400 cm^−1^ range. The morphological characteristics of nanoparticles were analyzed using FE-SEM (TESCAN, MIRA3 model, Warrendale, PA, USA). Nitrogen adsorption–desorption measurements determined the specific surface area (SBET) on a Nova 3200 gas-sorption system. The materials were vacuum-degassed for 20 h at 120 °C to evacuate any gas or humidity. The BJH method was applied to evaluate pore size distribution. Optical properties were analyzed through absorption spectra obtained using a Cary-5000 UV–Vis (Agilent Technologies, Santa Clara, CA, USA) spectrometer equipped with a polytetrafluoroethylene (PTFE) integration sphere in the 2000–200 nm range. A dynamic light scattering instrument (DLS, Microtrac Nanotrac Wave II, Montgomeryville, PA, USA) was used to calculate the average particle size, size distribution, and ζ-potential in water suspensions (1 mg/mL).

### 2.3. Antioxidant Activity

A 4 mg amount of DPPH was dissolved in 100 mL technical-grade ethanol, and absorbance was monitored in 1 mL quartz cuvettes using a Cary 60 UV–Vis spectrophotometer (Agilent Technologies, Santa Clara, CA, USA) at 517 nm. The mixture was kept under moderate stirring for 2 h. Then, 200 μL of DPPH solution was mixed with 20 mL of Z, ZS, ZL, and ZLS at 2.5, 5, 10, 20, 40, 80, and 160 μg/mL, respectively. Samples were maintained in a dark place for 30 min, and their absorbance was determined under the same conditions.

### 2.4. Analysis of Cytotoxicity

#### 2.4.1. Cell Lines Culture

Human breast adenocarcinoma MCF-7 cells (ATCC-HTB-22) and human colorectal adenocarcinoma HT-29 cells (ATCC-HTB-38) were acquired from the American Type Culture Collection (ATCC). These cells were cultivated at a temperature of 37 °C and a 5% CO_2_ concentration. The culture medium used was Dulbecco’s Modified Eagle’s Medium (DMEM), supplemented with 10% fetal bovine serum (FBS) from BenchMark, Gemini Bio Products (Woodland, CA, USA). The cell medium contained 1% penicillin–streptomycin (Sigma-Aldrich), 1% L-glutamine, and 1.5 g/L sodium bicarbonate (Sigma-Aldrich).

#### 2.4.2. MTT Assay

The cytotoxicity of Z, ZL, ZLS, and ZS on MCF-7 and HT29 cells was evaluated within a 96-well plate, where 10,000 cells per well were placed. These cells were seeded and allowed to adhere for a 24 h period in DMEM in a controlled environment of 37 °C and 5% CO_2_. Subsequently, the cell medium was removed, and different concentrations (2.5, 5, 10, 20, 40, 80, and 160 µg/mL) of Z, ZL, ZLS, and ZS nanoparticles were placed into the wells in a final volume of 100 µL of DMEM. Afterward, cells underwent a 24 h incubation phase at 37 °C and 5% CO_2_. Following the incubation period, the media were aspirated, and the cells were subjected to triple-washing with 200 µL of PBS 1×. The reduction of MTT (methyl-4,3-thiazolyltetrazolium), provided by Sigma-Aldrich, was used as an indicator cell viability. Briefly, 0.5 μg/μL of MTT was placed in each well alongside 90 μL of media, followed by incubation in darkness at 37 °C and 5% CO_2_ for 4 h. After this, 100 μL of isopropanol was added to each well and was incubated for 30 min at room temperature in the dark. The samples’ absorbances were measured using an ELISA plate reader (GoScan, Thermo Scientific, Waltham, MA, USA). To establish a more accurate baseline, the background absorbance of the cell viability test was gauged at 690 nm and subtracted from the absorbance values recorded at 570 nm. The assessment of cell viability involved a control group of cells cultured in DMEM without any treatment, while a positive control entailed exposing cells to 1% Triton X-100 to induce cell death. The absorbance values obtained from the positive control were employed to establish a baseline of 100% cell viability, enabling the depiction of results as the percentage of cell viability relative to Z, ZL, ZLS, and ZS concentration. The experiments were conducted independently in triplicate, each comprising internal triplicates for enhanced accuracy.

#### 2.4.3. ROS Generation Assay

Cells were seeded into a 96-well plate and incubated for 24 h at a temperature of 37 °C. During this time, varying Z, ZL, ZLS, and ZS concentrations (2.5, 5, 10, 20, 40, 80, and 160 µg/mL) were administered to each well of the plate. After this, the cells were subjected to a thorough triple-washing, utilizing 200 μL of PBS 1×. Subsequently, the cells were placed in the dark and treated with 2′,7′-dichlorofluorescein diacetate (25 μM) for 1 h at 37 °C and 5% CO_2_. The ensuing fluorescence was recorded with a Varioskan microplate reader (Thermo Scientific, Waltham, MA, USA) using an excitation laser operating at 485 nm and an emission laser operating at 530 nm.

### 2.5. Toxicity Evaluation In Vivo

The possible toxicity of Z, ZL, ZLS, and ZS was studied using the *A. salina* shrimp model. Briefly, dried cysts from *A. salina* were placed in a container with 35 g artificial sea salt dissolved in 1 L of distilled water. The container was maintained at 28–30 °C under vigorous aeration and constant illumination for 48 h. Once the nauplii hatched, 250 μL of nauplii specimens were placed per well in a 96-well plate together with Z, ZL, ZLS, or ZS at the following concentrations: 2.5, 5, 10, 20, 40, 80, and 160 μg/mL. The number of surviving nauplii was monitored for 48 h using an inverted microscope (Leica DMi1, Wetzlar, Germany) equipped with a FLEXACAM C1 camera through the Leica software version 3.3.0 (Leica Microsystems, MA, Germany). Experiments were performed in triplicate.

### 2.6. Statistical Analysis

Statistical analyses were performed using OriginPro 2023 software. To determine the cytotoxic effects of Z, ZL, ZLS, and ZS on the production of reactive oxygen species (ROS) and antioxidant activity, an analysis of variance was carried out together with the comparison of means by the Tukey test, setting a confidence level of 95%. Each experiment was performed in triplicate.

### 2.7. Machine Learning Modeling

With the data obtained from the experiments conducted in the synthesis and material characterization phases, machine learning models were generated to try to establish a function that connects parameters with the antioxidant activity (AA), measured as an independent variable relevant to this work. A dataset of nine independent variables was formed: Material, Gs, TC, EG, Defects, Charge, DLS, Method, and Concentration. AA was taken as the dependent variable. In total, 196 observations were used. Two preprocessing operations were applied to the original dataset.

The first is an operation known as the one-hot encoder (OHE). One-hot encoding is a technique used in data processing and machine learning to represent categorical variables, such as categories or labels, in a numerical form suitable for machine learning models. It creates a new binary column (0 or 1) for each unique category in the original categorical variable. Each binary column represents the presence or absence of a particular category, thus converting the categorical variable into a set of numerical features that machine learning algorithms can interpret. This technique helps avoid misinterpretations of numerical relationships between categories and allows models to adequately capture categorical variable information in their predictions. OHE was applied to the columns coding for the “material” and “method” variables in this case. The first contained four different values, and the second contained three, which means that, instead of those two initial variables, we will have seven in the transformed dataset. The total number of transformed independent variables (features) will be 14.

The second preprocessing operation was scaling. Scaling is an essential step in data preprocessing that involves transforming the numerical features of a dataset so that they have a common and comparable scale. This is usually achieved by normalizing or standardizing the features, which means adjusting their values to be in a specific range. Normalization usually scales features to be within the interval [0, 1], while standardization transforms them into a mean of 0 and a standard deviation of 1. The main goal of scaling is to prevent differences in feature scales from negatively affecting machine learning algorithms, as such differences may influence some algorithms in orders of magnitude, leading to misinterpreted relationships between features and, ultimately, to less accurate models. The original dataset is separated into two sets, train and test, comprising 70% and 30% of the total, respectively. As their names indicate, the former trains the models, and the latter evaluates them.

Model training and evaluation: In the first experiment, eight regression models were trained: linear regression (LR), multilayer perceptron (MLP), random forest (RF), decision trees (DTs), extremely random trees (ETs), k-nearest neighbors (KNNs), gradient boosting (GB), and support vector regressor (SVR).

The model with the best results is usually adjusted by modifying its hyperparameters, and, finally, an analysis of feature importance is performed. In this case, the optimized model is used. Feature importance is determined through a process that provides information such that it is possible to identify the most critical features of the model, i.e., those that are the most relevant to the model. This can help reduce the dimensions in the model and improve its interpretability. Feature importance helps engineers and scientists gain some insight into the underlying relationships in the data. In addition, if less critical features are removed from the model, the model’s performance can be improved. All experiments were run on an HPZ440 Server with a Xeon E51620V3 Processor at 3.5 GHz, 16 GB RAM, 4 cores, and 8 processers running Ubuntu 22.04 and Python 3.11 with SciKit-Learn and Numpy libraries.

## 3. Results and Discussion

### 3.1. Characterization of Nanomaterials

**Figure 1:**
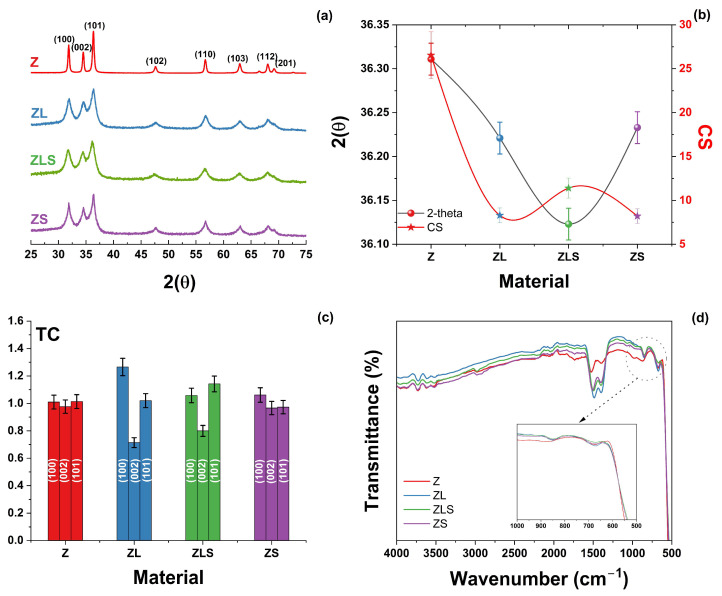
(**a**) XRD patterns, (**b**) the shift in the 2(q) position and average crystallite size, (**c**) TC analysis, and (**d**) FTIR evaluation of Z, ZL, ZLS, and ZS materials.

The effect of the incorporation of lanthanide elements in the crystal structure of ZnO was analyzed via X-ray diffraction. Figure 1a provides the XRD results of the (La, Sm)-doped ZnO nanoparticles. All the samples exhibited reflections that correspond to the (100), (002), (101), (102), (110), (103), (112), and (201) planes and were matched with the hexagonal wurtzite structure of ZnO (JCPDS # 36-1451). No secondary phases related to La^3+^ or Sm^3+^ were observed, including at high doping contents. It is evident that the incorporation of the lanthanide elements decreased the crystallinity of the materials.

A shift in the diffraction peaks was evident for the ZLS sample (see Figure 1b). The lattice constants *a* and *c* and the different structural parameters were calculated from the XRD analysis. The results are shown in Table 1, where standard deviation is presented between parenthesis, and cell volume is depicted as (Å)^3^. Only the ZLM sample exhibits an increase in the lattice parameters (after cell refinement using MDI Jade Software version 6.0). This effect can be due to the differences in the ionic radii of Zn^2+^ (0.74 Å), La^3+^ (1.16 Å), and Sm^3+^ (0.96 Å) [23]. Some reports have shown that the face orientation of ZnO can improve its activity for H_2_O_2_ generation [24]. The crystallinity and high proportion of polar planes of the ZnO structure are fundamental for photocatalytic reactions. Polar planes such as the Zn-terminated (001) and O-terminated (001) planes promote the formation of ROS [25].

The texture coefficient (TC) was calculated to evaluate the preferential crystallite orientation of Z, ZL, ZLS, and ZS. The results are shown in Figure 1c. The ZL and ZLS samples exhibit significant differences in their principal plane TC values. The results of the FTIR analysis of the undoped and (La, Sm)-doped ZnO nanoparticles are presented in Figure 1d. All samples exhibited similar FTIR spectra, with characteristic vibrational bands in the 3320, 1560–1500, 1400–1300, 1020–930, and 680–500 cm^−1^ regions, which correspond to -OH (stretching mode), H-O-H (bending vibration), C-O (stretching vibration), O-H (asymmetric stretching), and Metal-O (stretching), respectively [26]. A slight shift in the position and intensity of the peak was observed after doping.

**Figure 2:**
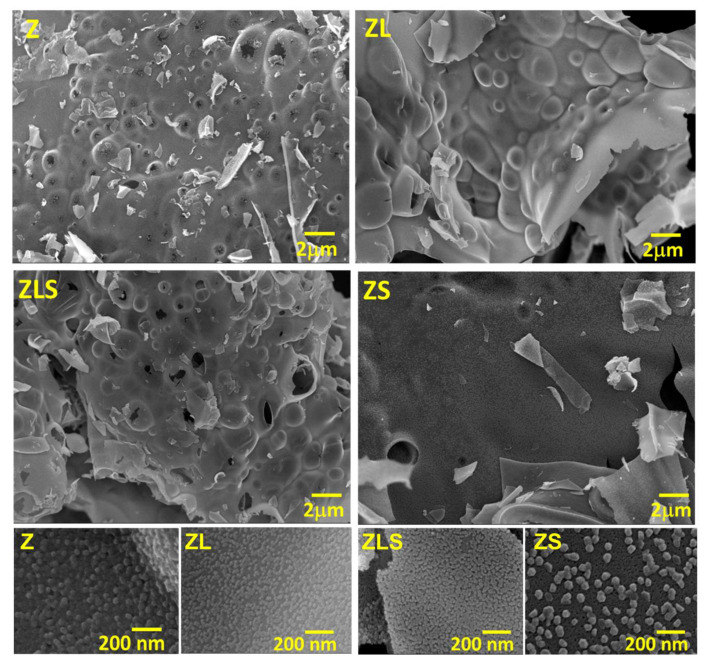
SEM observation of Z, ZL, ZLS, and ZS and the effect of the ultrasound-assisted synthesis route in their microstructure at low and high magnification.

The effect of the ultrasound on the microstructure of Z, ZL, ZLS, and ZS was analyzed by SEM. Figure 2 compiles the SEM analyses of samples at low and high magnification. From the low-magnification images, it can be seen that the microstructure consists of interconnected bubbles. The PVA-sucrose reaction mechanism was discussed in a previous work [27]. However, a polymeric resin is formed in the final reaction, which allows for a laminar-type microstructure to form. These images show that most of the bubbles have collapsed. In addition, small laminar fragments of materials can be observed. The high-magnification images show that all of the materials are entirely nanostructured.

**Figure 3:**
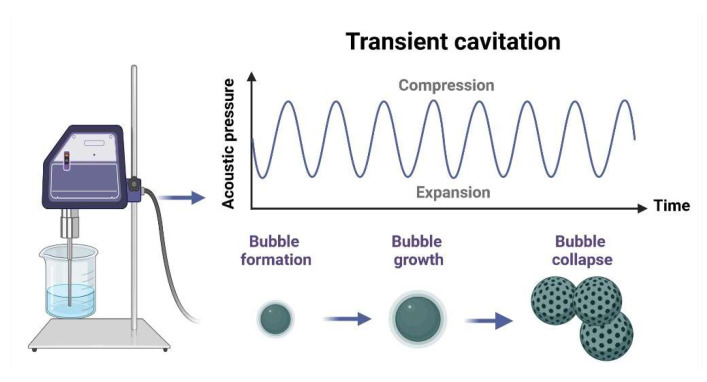
Schematic representation of cavitation bubbles displaying transient cavitation.

It is well understood that sonochemically assisted synthesis induces a change in the microstructure of the materials due to physical and chemical processes. In the initial steps, cavitation bubbles are formed during nucleation. Then, the cavitation bubbles change to stable or transient cavitation. Transient cavitation occurs when bubbles exceed their equilibrium size and collapse [28]. When bubbles collapse, shock waves and shear forces are produced, carrying a lot of energy with them [29]. According to the literature, when the sonication time exceeds 20 min, there is a negative effect on the particle size and uniformity resulting from Ostwald’s process [30]. This process is depicted in Figure 3.

**Figure 4:**
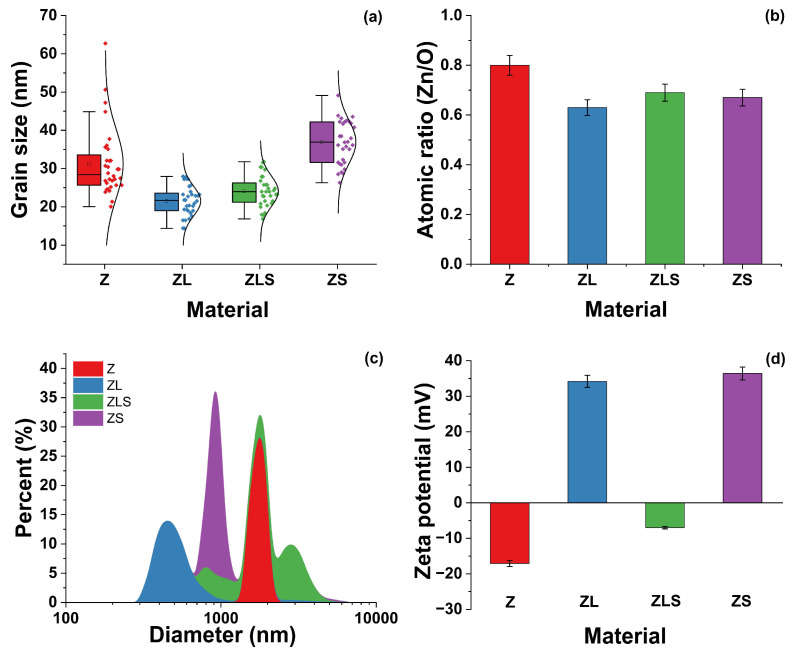
(**a**) Grain size particle distributions, (**b**) atomic ratio (Zn/O) from EDS measurements, (**c**) size distribution analysis, and (**d**) ζ-potential value of Z, ZL, ZLS, and ZS.

The grain size distribution of each sample was investigated, and the results are presented in Figure 4a. In this regard, the average grain size is 31, 21, 24, and 37 ± 5% nm for Z, ZL, ZLS, and ZS, respectively. A single-factor analysis of variance (ANOVA) and comparison of means using a Tukey test were performed with a 95% confidence level to determine if differences in grain size were obtained. The ANOVA results determined significant differences in the grain size, with a *p*-value < 0.05. However, not all materials were different when compared to each other; that is, there are differences between all materials, except for between ZLS and ZL. Usually, the grain size is related to ROS formation.

EDS () was utilized to analyze the elemental composition of all the samples. All the EDS spectra exhibit significant Zn and O peaks, confirming the presence of these elements. In the same regard, representative peaks from La^3+^ and Sm^3+^ were visible in the doped samples. The carbon content was not considered for any of the samples because the samples were fixed to carbon tape for analysis. The EDS results showed variations in the Zn/O ratio, which decreased as doping increased, suggesting structural defects (see Figure 4b). Regarding their analysis via DLS, it can be observed in Figure 4c that ZnO-NPs comprise particles that range from 1406 to 1936 nm, whereas the size of ZL and ZLS encompass NPs ranging in size from 332 to 868 nm and 750 to 5040 nm, respectively. The size distribution of ZS ranges from 739 to 1040 nm. The average sizes of Z, ZL, ZLS, and ZS are 1815, 1766, 2930, and 454 nm, respectively. Figure 4d shows that the ζ-potential values of Z, ZL, ZLS, and ZS are −17.1, 34.2, −7.0, and 36.0 mV, respectively.

**Figure 5:**
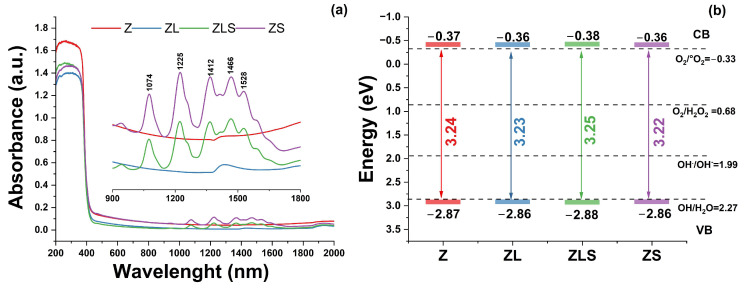
(**a**) Absorbance spectra and (**b**) optical band gap, conduction, and balance band values (CB, VB) of Z, ZL, ZLS, and ZS.

The absorbance spectra and optical parameters of the (La, Sm)-doped ZnO nanoparticles are depicted in Figure 5. From the absorbance spectra (Figure 5a), it can be seen that all the samples showed an absorption edge around 370 nm, which corresponds to the direct band gap of ZnO [31]. The inset of Figure 5a shows a magnification in the 900 to 1800 nm range of the absorbance spectra. A band placed at 1390 nm related to hydroxyl groups was observed in the Z and ZL samples. The ZLS and ZS samples exhibited several bands whose intensities increased as the Sm^3+^ content increased. These bands are placed around 1592, 1528, 1466, 1412, 1370, 1225, and 1074 nm. These bands are characteristic of Sm^3+^-activated materials. These optical transitions are from the ^6^H_13/2_, (^6^F_3/2_, ^6^H_15/2_, ^6^F_1/2_), ^6^F_5/2_, ^6^F_7/2_, and ^6^F states [32]. The optical band gap (*E_g_*) was calculated using the Kubelka–Munk function and Tauc’s plot from the absorbance spectra. The results are shown in Figure 5b. No significant variations in the *E_g_* values were observed. In addition, the conduction band (CB) and valence band (VB) values related to ROS formation were calculated.

### 3.2. Antioxidant Activity

**Figure 6:**
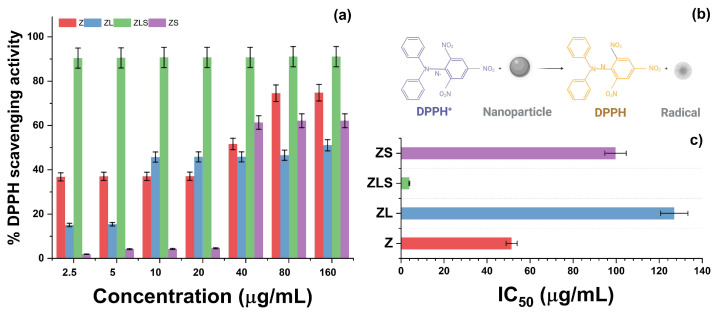
(**a**) DPPH scavenging activity, (**b**) schematic representation of the antioxidant activity of nanoparticles, and (**c**) IC_50_ values of Z, ZL, ZLS, and ZS.

Antioxidants are classified into either synthetic or natural compounds. Synthetic antioxidants refer to a representative class of nanometric structures that can inhibit the generation of free radicals. Oxide, metal-based, and functionalized nanoparticles belong to this category. The antioxidant activities of Z, ZL, ZLS, and ZS were determined via the DPPH method, and are presented in Figure 6. Initially, it can be noted that the treatment with 2.5 μg/mL of Z resulted in the inhibition of DPPH radicals by 34.483 ± 1.980%, whereas treatment with 5, 10, and 20 μg/mL scavenged 35.329 ± 3.447, 37.490 ± 3.250, and 37.225 ± 4.644% of the DPPH radicals, respectively. At higher concentrations, the antioxidant activity of Z nanoparticles increased.

For instance, the 40 μg/mL treatment inhibited the formation of 46.196 ± 13.568% of DPPH radicals. At 80 and 160 μg/mL, 70.267 ± 8.276 and 76.042 ± 1.167% of DPPH radicals were scavenged, respectively. The antioxidant activity of the developed nanomaterials varied with the presence of La^3+^. As observed in the same figure, treatment with ZL at 2.5 and 5 μg/mL resulted in the scavenging of 13.821 ± 1.469 and 16.385 ± 1.151% of DPPH radicals, respectively. Moreover, treatment with 10 μg/mL of ZL inhibited the generation of radicals by 46.151 ± 0.380%. However, the proportion of scavenged DPPH radicals following treatment with 20 or 40 μg/mL of ZL remained similar: 46.265 ± 0.438 and 46.462 ± 0.463%, respectively. At 80 and 160 μg/mL, treatment with ZL scavenged 46.709 ± 0.341 and 49.226 ± 1.653% of DPPH radicals, respectively. The antioxidant activity of this series was enhanced with the addition of Sm^3+^.

The treatment with 2.5, 5, 10, 20, and 40 μg/mL of ZLS decreased the generation of DPPH radicals by 90.421 ± 0.880, 90.497 ± 0.522, 90.534 ± 0.095, and 90.711 ± 0.151%, respectively. In the same context, treatment with 80 μg/mL scavenged 90.762 ± 0.344% of DPPH radicals, whereas treatment with 160 μg/mL inhibited 90.784 ± 0.238% of free radicals. On the other hand, treatment with 2.5, 5, 10, and 20 μg/mL of ZS inhibited the following proportions of DPPH radicals: 2.756 ± 2.807, 4.064 ± 0.202, 4.447 ± 0.238, and 5.293 ± 1.040%, respectively. Interestingly, the antioxidant activity of ZS increased at 40 μg/mL and resulted in 58.612 ± 6.433% DPPH radicals being scavenged. At 80 and 160 μg/mL, the percentage of scavenged DPPH radicals was 60.495 ± 0.785 and 62.161 ± 0.491%, respectively. The half-maximum inhibitory concentration values (IC_50_) of Z, ZL, ZLS, and ZS against DPPH radicals were 51.140 ± 14.985, 126.948 ± 5.811, 3.898 ± 0.122, and 99.701 ± 8.262 μg/mL, respectively (Figure 6c). As depicted in Figure 6b, the antioxidant activity of these nanomaterials can be attributed to the electron donation of the oxygen atom from ZnO to the odd electron from the nitrogen atom located in the chemical structure of DPPH.

There are various reports on the antioxidant activity of ZnO-NPs synthesized through different approaches. For example, it was found that ZnO-NPs biosynthesized with *Achillea nobilis* extract can scavenge 5–60% of DPPH radicals at concentrations ranging from 20 to 1000 ppm [33]. In another study, ZnO nanoflowers synthesized via the hydrothermal and precipitation methods inhibited the generation of 52.7–60.61% and 55.63–64.29% of DPPH radicals, respectively [34]. In contrast, treatment with 10 μL of ZnO-NPs prepared via the coprecipitation method combined with exposure to different light regimens inhibited 5.56–28.78% of DPPH radicals [35].

The scientific evidence regarding the antioxidant activity of La-based nanostructures is limited. For example, it has been reported that LaNPs synthesized with the aqueous extract from *Moringa oleifera* can scavenge 61.0 and 78.5% of DPPH radicals at 100 and 200 μg/mL, respectively [36]. Using other experimental models, the capacity of LaNPs to prevent the generation of free radicals has been related to the electronic configuration of their 4f shielded electrons [37]. For Sm-based nanomaterials, however, biomedical evidence on their antioxidant activity is scarce. However, their capacity to disrupt the activities of enzymes involved in the antioxidant systems of plants has been documented [38]. The results presented in this work are challenging to compare because of the variabilities in synthesis techniques and their experimental conditions, in the models used to evaluate their antioxidant activity, in the utilized concentrations, and in the presentation of results.

### 3.3. Cytotoxic Activity and ROS Assay

Cancer is the term encompassing a group of diseases characterized by the abnormal proliferation and growth of cells [4]. In contrast to healthy cells, cancer cells possess aberrations in their genome that result in sustained proliferative signaling, evasion of growth suppressors, replicative immortality, escape from immune response cell invasion and metastasis, the capacity for angiogenesis, resistance to cell death, and deregulated cellular metabolism.

**Figure 7:**
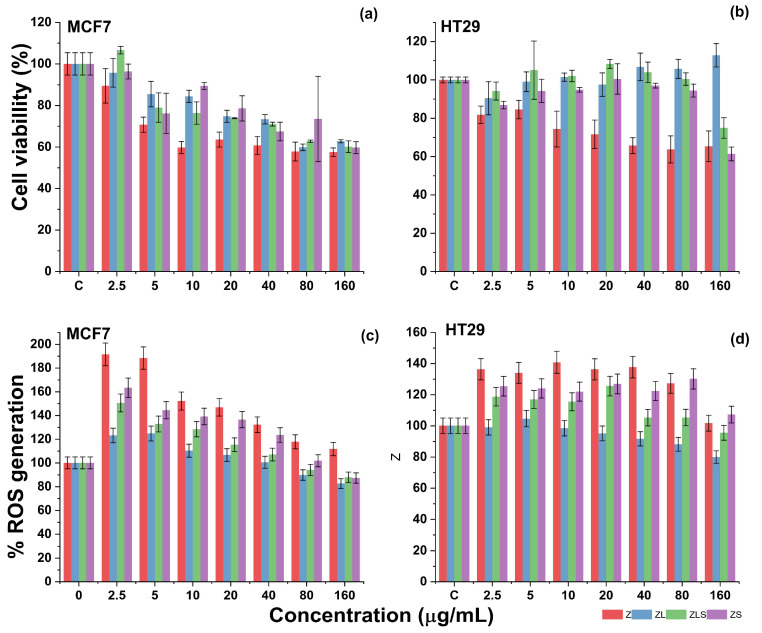
Effect of Z, ZL, ZLS, and ZS in the generation of ROS in the MCF-7 and HT29 cell lines.

The cytotoxicity of Z, ZL, ZS, and ZLS was evaluated against two cell lines: MCF-7 and HT29 (see Figure 7). The former is a representative cellular model used to explore the potential use of nanostructures against breast cancer [39], which is the most common cancer diagnosed among women worldwide, and the fifth most common cause of cancer-related deaths in the last few years [40]. Similarly, the latter is utilized to investigate the possible use of nanomaterials to develop therapies against colorectal cancer [41], which is a complex type of cancer that has remained as the third most common type of cancer diagnosed for men and women worldwide and the third most common cause of cancer-related deaths in the United States of America (USA) [42].

Against the MCF-7 cell line, the cytotoxicity of Z increased in a dose-dependent manner. As shown in Figure 7a, treatment with 2.5, 5, and 10 μg/mL of Z resulted in 89.440 ± 8.306, 70.683 ± 3.662, and 59.689 ± 2.948% cell viability, respectively. Regarding cell death, treatment at those concentrations caused the death of 10.559 ± 8.306, 29.316 ± 3.662, and 40.310 ± 2.948% of MCF-7 cells. At 20, 40, 80, and 160 μg/mL, treatment with Z led to 63.540 ± 3.598, 60.683 ± 4.307, 57.763 ± 4.518, and 57.453 ± 2.044% MCF-7 cell viability.

In terms of cell death, these values represent the death of 36.459 ± 3.598, 39.316 ± 4.307, 42.236 ± 4.518, and 42.546 ± 2.044% of MCF-7 cells, respectively. In the same figure, it can be noted that treatment with 2.5, 5, and 10 μg/mL ZL resulted in 65.714 ± 6.907, 85.465 ± 6.101, and 84.347 ± 2.912% MCF-7 cell viability. In contrast, 20, 40, 80, and 160 μg/m of ZL resulted in 74.782 ± 2.901, 73.354 ± 2.223, 59.813 ± 1.592, and 62.732 ± 0.753% cell viability, respectively. Expressed as cell death, these values constitute the cell death of 4.285 ± 6.907 (2.5 μg/mL), 14.534 ± 6.101 (5 μg/mL), 15.652 ± 2.912 (10 μg/mL), 25.217 ± 2.901 (20 μg/mL), 26.645 ± 2.223 (40 μg/mL), 37.267 ± 0.753 (80 μg/mL), and 40.186 ± 1.592% (160 μg/mL) of MCF-7 cells. Interestingly, the incorporation of Sm^3+^ into ZL did not enhance its cytotoxicity.

For example, MCF cells proliferated at 2.5 μg/mL, resulting in 106.64% cell viability. However, the cytotoxicity of ZLS was evident at 5, 10, and 20 μg/mL which resulted in 78.944 ± 7.113, 76.335 ± 5.408, and 73.850 ± 0.215% MCF-7 cell viability. The recorded cell viability at 40, 80, and 160 μg/mL were 70.993 ± 0.931, 62.732 ± 0.569, and 60.124 ± 2.803%, respectively. These findings can be also expressed as the cell death of 21.055 ± 7.113 (5 μg/mL), 23.664 ± 5.408 (10 μg/mL), 26.149 ± 0.215 (20 μg/mL), 29.006 ± 0.931 (40 μg/mL), 37.267 ± 0.569 (80 μg/mL), and 39.875 ± 2.803 (160 μg/mL) of cancer cells. Regarding the cytotoxicity of ZS, it can be observed in Figure 7a that treatment with 2.5–160 μg/mL resulted in the viability of 96.33–59.68% of MCF-7 cells (3.66–40.31% cell death). The cytotoxicity of Z, ZL, ZLS, and ZS was distinct against HT29 cells, a representative model of human colon cancer cells.

As observed in Figure 7b, the cytotoxicity of Z against HT29 cells increased concerning the used concentrations. Initially, treatment with 2.5 μg/mL caused the death of 18.230 ± 4.519% of HT29 cells, whereas 5, 10, and 20 μg/mL of Z resulted in 15.516 ± 4.779, 25.66 ± 9.353, and 28.37 ± 7.436% cell death. For Z, the highest cytotoxicity was recorded at 40, 80, and 160 μg/mL, as these doses caused the cell death of 34.336 ± 4.162, 34.690 ± 8.017, and 36.283 ± 7.079% of HT29 cells. The treatment with ZL against the HT29 cell line in the suggested concentrations was determined to not be cytotoxic since a significant decrease in cell viability was not observed. This phenomenon was similar during treatment with ZLS, that is, until the treatment with 160 μg/mL ZLS, which resulted in the death of 25.073 ± 5.386% of HT29 cells. Comparably, treatment with ZS was poorly cytotoxic against the HT29 cell line, as the highest cytotoxicity was observed at 160 μg/mL (38.643 ± 3.593% cell death).

Given the results obtained against the MCF-7 and HT29 cell lines, the median lethal concentration (LC_50_) values were calculated and are compiled in Table 2. Against the MCF-7 cell line, Z, ZL, ZLS, and ZS are moderately cytotoxic, as indicated by their LC_50_ values: 161.418 ± 17.660, 181.885 ± 9.455, 174.982 ± 13.250, and 200.835 ± 57.778 μg/mL, respectively. In contrast, their activity against the HT29 cell line is considered weak, again indicated by their LC_50_ values: 249.985 ± 93.527 (Z), 504.917 ± 161.917 (ZL), 390.440 ± 116.376 (ZLS), and 260.919 ± 24.677 μg/mL (ZS). This is in accordance with the classification from the National Cancer Institute (NCI) from the USA [43].

The anti-cancer activity of the developed nanomaterials can mainly be attributed to the presence of Zn^2+^. However, in healthy cellular models, recent studies have demonstrated that treatment with ZnO-NPs did not have a significant effect on the viability of human gingival fibroblasts (HGF-1) nor did it change their morphology at concentrations ranging from 5 to 25 μg/mL [44]. Comparably, in another study, treatment with 1 and 5 μg/mL of ZnO-NPs prepared via a wet-chemical route did not decrease the viability of HFGs or human umbilical vein endothelial cells (HUVECs) [45]. In fact, treatment with ZnO-NPs significantly enhanced their metabolic activity. In the case of the effect of La^3+^-based nanomaterials, phosphate glasses doped with distinct contents of La_2_O_3_ NPs have been evidenced as biocompatible materials since treatment with 2.5 or 5 mg did not significantly reduce the viability of fibroblasts derived from baby hamster kidneys (BHKs) [46]. Similarly, Sm^3+^-doped hydroxyapatite coatings have been reported to not compromise the viability or morphology of the HGF-1 cell line [47]. In the synthesis of nanocomposites, Sm_2_O_3_, together with Cr_2_O_3_, graphene oxide, and polycaprolactone, did not reduce the cell viability of the human skin cell line HFb-4 at 2.5, 5, or 19.5 μg/mL, respectively [48].

**Figure 8:**
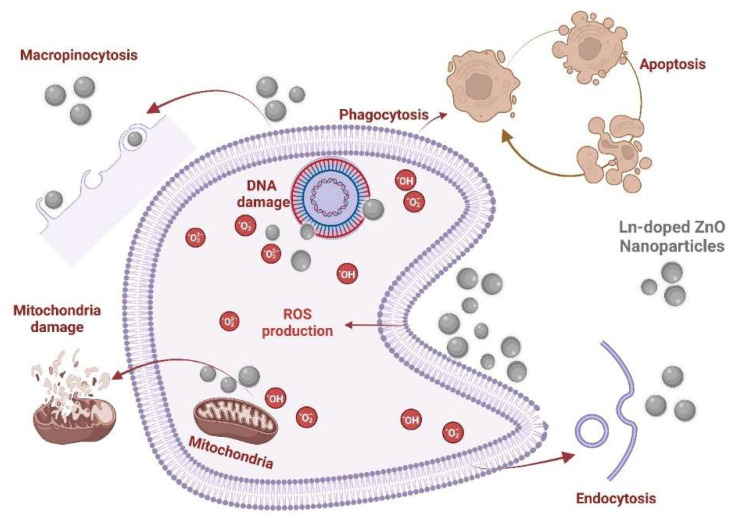
Schematic representation of the possible anti-cancer mechanisms of (La, Sm)-doped ZnO nanoparticles.

As shown in Figure 8, the cytotoxicity of ZnO-NPs initiates upon their cellular entry, mediated by endocytosis, micropinocytosis, or phagocytosis [49,50]. The cellular uptake of ZnO-NPs can occur in the intracellular release of Zn^2+^ ions, resulting in cell death by zinc-dependent protein activity disequilibrium [51]. Once entered into the intracellular environment, ZnO-NPs can upregulate the generation of ROS levels, promote oxidative stress, damage genetic material (DNA), induce the activation of caspases (e.g., caspase 3) via intrinsic mitochondrial routes, or alter the functionality of mitochondria by compromising its membrane potential [52]. Even though evidence about the possible anti-cancer mechanisms of La^3+^ or Sm^3+^ remains limited, it has been documented that La_2_O_3_-NPs can exert cytotoxic effects against glioblastoma cells through a multifaceted phenomenon that includes enhanced intrinsic and extrinsic apoptosis, the upregulation of ROS levels, direct DNA damage, and autophagy induction [53]. When used for doped ZnO-NPs and combined with UV light, it can also enhance the generation of ROS. In comparison with other doping elements such as Eu^3+^ and Gd^3+^, ZnO-NPs doped with Sm^3+^ have been demonstrated to elevate apoptotic biomarkers (i.e., Bax) and arrest the cell cycle of Ehrlich ascites carcinoma cells at the G2 phase in mice bearing Ehrlich solid tumors [54].

Under healthy physiological conditions, reactive radicals such as ROS are required to maintain cell homeostasis or regulate cell signaling pathways involved in cell metabolism, differentiation, and proliferation [55]. In pathological processes, the overgeneration of free radicals can cause the development of metabolic syndromes, respiratory and cardiovascular diseases, and cancer [56]. Since treatment with ZnO-NPs tends to affect the levels of ROS, the capacity of Z, ZL, ZLS, and ZS to enhance or decrease their generation was evaluated against the MCF-7 and HT29 cell lines. As depicted in Figure 7c, MCF-7 cells treated with Z at 2.5, 5, and 10 μg/mL exhibited enhanced levels of ROS by 191.448 ± 16.388, 188.313 ± 12.370, and 152.074 ± 14.459%, respectively. In comparison, the treatment of MCF-7 cells with Z at 20, 40, 80, and 160 μg/mL resulted in downregulated levels of ROS of 146.792 ± 4.923, 132.139 ± 17.328, 117.747 ± 10.580, and 111.608 ± 17.724, respectively. Comparably, MCF-7 cells treated with ZL at 2.5, 5, and 10 μg/mL presented 123.051 ± 5.106, 124.730 ± 5.706, and 110.236 ± 3.919% reduction in ROS generation, respectively. At 20, 40, 80, and 160 μg/mL, the generation of ROS decreased in the following proportions: 106.623 ± 5.757, 100.427 ± 4.275, 89.701 ± 1.180, and 82.543 ± 1.834. Regarding the capacity of ZLS to induce the generation of ROS, it was determined that the levels of ROS increased at 2.5 (150.484 ± 7.717%), 5 (132.748 ± 8.896%), and 10 μg/mL (128.410 ± 4.888%), whereas they decreased at 20 (115.262 ± 9.931%), 40 (107.003 ± 6.473%), 80 (93.975 ± 4.717%), and 100 μg/mL (87.799 ± 1.319%). Finally, treatment with 2.5, 5, and 10 μg/mL of ZS increased the generation of 163.304 ± 11.732, 144.409 ± 16.712, and 139.133 ± 16.837% ROS, respectively, and decreased their formation at 20 (136.425 ± 8.471%), 40 (123.423 ± 4.996%), 80 (101.755 ± 5.611%), and 160 (87.219 ± 5.760%) μg/mL, respectively.

As observed in Figure 7d, treatment with 2.5, 5, and 10 μg/mL of Z enhanced the generation of ROS in the HT29 cell line by 136.315 ± 1.406, 133.941 ± 7.742, and 140.773 ± 3.069, respectively. As the concentration of Z increased to 20, 40, 80, and 160 μg/mL, the levels of ROS generation decreased as follows: 136.293 ± 7.609, 137.650 ± 8.022, 127.244 ± 9.814, and 101.694 ± 2.658% ROS generation. Similarly, the generation of ROS during the treatment with 2.5 μg/mL of ZL increased by 98.972 ± 2.354%. In comparison, the levels of ROS following treatment with 5, 10, and 20 μg/mL of Z resulted in 104.550 ± 8.745, 98.413 ± 4.799, and 95.104 ± 1.709% increases. Comparable activity was recorded at 40, 80, and 160 μg/mL as 91.621 ± 2.065, 88.066 ± 3.269, and 79.909 ± 2.471% increases in ROS levels were registered, respectively. The treatment of ZLS at 2.5, 5, 10, and 20 μg/mL promoted the generation of 118.665 ± 3.898, 116.846 ± 9.866, 115.414 ± 8.827, and 125.513 ± 26.728% increases in ROS levels, respectively. However, these levels decreased at 40 (105.238 ± 4.810%), 80 (105.293 ± 8.144%), and 160 (95.471 ± 5.463%) μg/mL. The treatment with ZS at 2.5, 5, 10, and 20 μg/mL caused the generation of ROS to increase by 125.410 ± 3.961, 123.991 ± 1.981, 121.893 ± 2.796, and 126.880 ± 2.680%, respectively. On the other hand, 40, 80, and 160 μg/mL of ZS resulted in 122.282 ± 2.569, 130.079 ± 9.940, and 107.189 ± 0.849% decreases in ROS levels.

It has been documented that the overproduction of ROS can lead to their accumulation within cancer cells, where they can cause damage to organelles, proteins, and genetic material and induce cell death [57]. Such overproduction was observed predominantly during treatment with Z, ZL, ZLS, and ZS against the MCF-7 cell line, specifically at 2.5, 5, or 10 μg/mL. However, as the concentration of the nanomaterials increased from 20 to 160 μg/mL, the levels of ROS were downregulated. This phenomenon can be related to the high antioxidant activity of the synthesized nanomaterials, which was demonstrated during the DPPH assay and documented for antioxidants that can diminish the generation of ROS and induce the apoptosis of cells from other cancer lines [58].

Taking the antioxidant activity and influence of Z, ZL, ZLS, and ZS in the generation of ROS together, the statistical analyses revealed significant differences associated with the treatment, concentration, and cell line, as well as their interactions, with a *p*-value < 0.05 for Z and ZS nanoparticles towards the evaluated cell lines. Lanthanum doping in the ZL and ZLS series was found to decrease antioxidant activity and reduce ROS production, showing no significant differences compared to the control treatment. In contrast, the Z and ZS series exhibited higher ROS production at the lowest concentrations studied. Significant variations were also observed between the two cell lines, with MCF7 demonstrating higher ROS production when exposed to the nanoparticle series. This trend persisted across concentrations, with 2.5 μg/L being the most influential for the treatments. Regarding toxicity, it was observed that all the nanoparticles evaluated showed some degree of toxicity compared to the control. The ZS series presented increased cell proliferation at the lowest tested concentration. Regarding antioxidant activity, the highest concentrations were associated with the most favorable effects, with the Z series consistently outperforming the control treatment across all evaluated concentrations.

### 3.4. Toxicity Evaluation In Vivo

The field of integrated nanomedicine and nanotoxicology is devoted to studying the possible adverse effects of nanomaterials on ecosystems and complex organisms. The toxicity of nanomaterials can be related to distinct factors such as impurities, surface features (e.g., the presence or absence of ligands and surface charges), size, and shape [59]. Among in vivo models used to evaluate the toxicity of potential bioactive structures or compounds such as plant extracts, natural products, and nanostructures, *A. salina* (a 1 mm marine invertebrate also known as the sea monkey) has been widely used [60].

**Figure 9:**
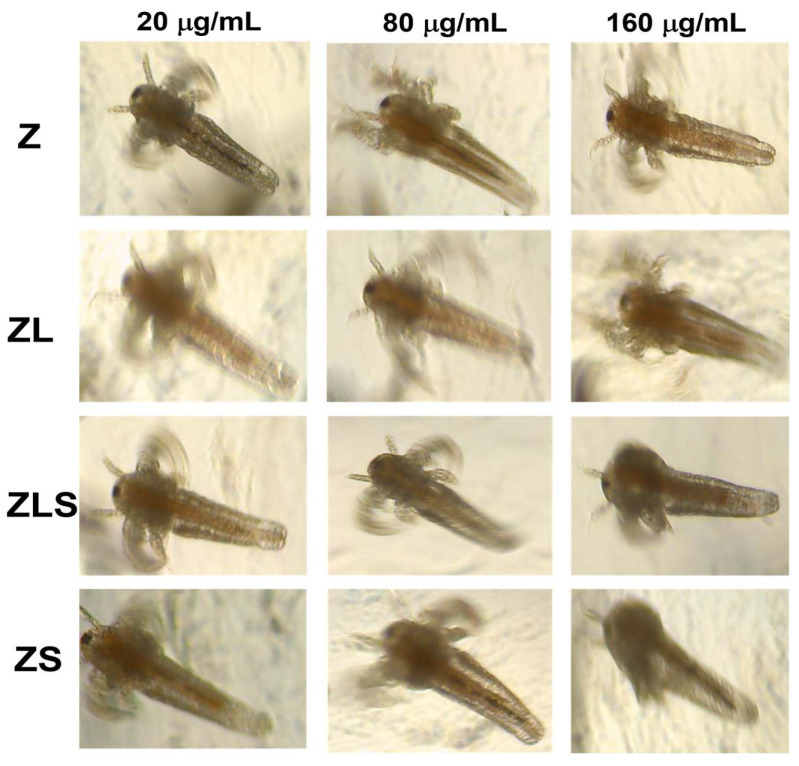
Effect of Z, ZL, ZLS, and ZS against *A. salina* nauplii.

Recently, it has been demonstrated that 40–60 nm ZnO-NPs can accumulate within *A. salina* nauplii and, hence, induce their death after 24 h in a dose-dependent manner (0.2–50 mg L^−1^) [61]. Comparably, it has been reported that treatment at <6.25 μg/ mL of ZnO-NPs synthesized with rutin presented a nauplii mortality rate of 30% after 8 h of exposure [62]. On the other hand, ZnO nanocolloids substituted with La^3+^ (La_0.1_Zn_0.9_O and La_0.2_Zn_0.8_O; −41.4 and −32.4 mV) synthesized via a modified sonochemical technique have been shown to induce the death of 10–60% of *A. salina* nauplii at 10, 100, and 1000 μg/mL [63]. However, the use of *A. salina* to test the toxicity of Sm-based nanomaterials has not been reported yet. The effect of Z, ZL, ZLS, and ZS on *A. salina* nauplii is presented in Figure 9. As shown in this figure, it can be observed that Z and ZS at concentrations of 20, 80, and 160 μg/mL accumulated along the gut of *A. salina* nauplii but did not lead to their death. In contrast, ZL and ZLS did not exert this effect nor did it induce morphological changes. The differences between these findings and other results can be due to variabilities in the size, shape, surface charge, synthesis route, and evaluated concentrations. The scarce toxicity of the developed nanomaterials against the proposed in vivo model suggests their biocompatibility while exerting anti-cancer and antioxidant properties at the mentioned concentrations.

### 3.5. Machine Learning Modeling

**Figure 10:**
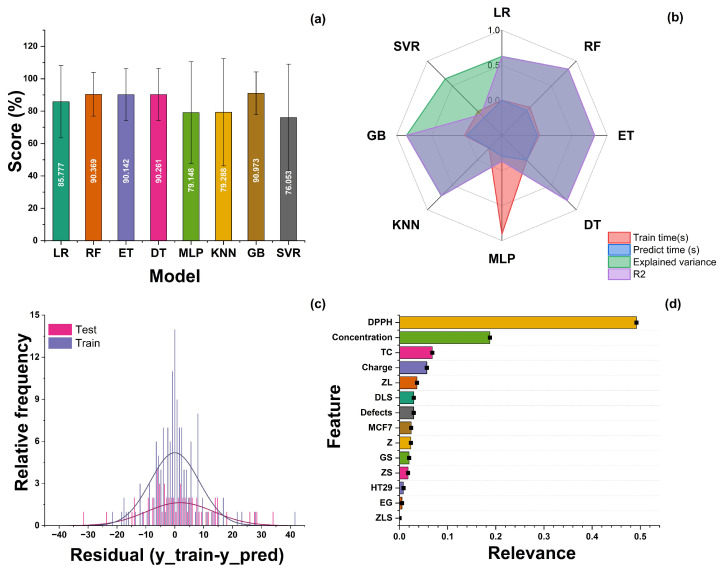
(**a**) Accuracy achieved by all the models after initial training, (**b**) other training parameters evaluated (training time, explained variance, and *R*^2^ coefficient), (**c**) distribution of the residuals when applying the GB model to both the train and test datasets, and (**d**) features sorted according to their relevance in the model.

From these data, various models were tested as regressors since the variable AA is numerical and continuous. Figure 10 summarizes the computational experiments performed. There are three main metrics used in the evaluation of regression models: R-squared (R2 or R^2^), mean squared error (MSE), and mean absolute error (MAE). In the case of the experiments developed in this work, two of them were calculated for each model (R2 and MAE). Figure 10a shows the values returned by the Sklearn score function in Python, which is based on the coefficient of determination R2. This value is calculated as follows: let u = sum ((y_test − y_predicted)2), and v = sum((y_test − y_test.mean())2); then, score = 1 − (u/v). This score is shown as a percentage in Figure 10a. A larger percentage indicates a better fit between the prediction and the true value. Figure 10b shows the training time, prediction time, explained variance, and correlation coefficient (*R2*) obtained for each model. The distribution of the residuals when applying the GB model to both the train and test datasets is shown in Figure 10c. Additionally, another common value to compare regression results is the root mean square error (RMSE), also called standard error, which corresponds to the following values for the models computed: LR: 22.28; RF: 13.43; ET:16.12; DT: 16.07; MLP: 31.4; KNN: 33.09; GB: 13.19; and SVR: 32.93.

**Figure 11:**
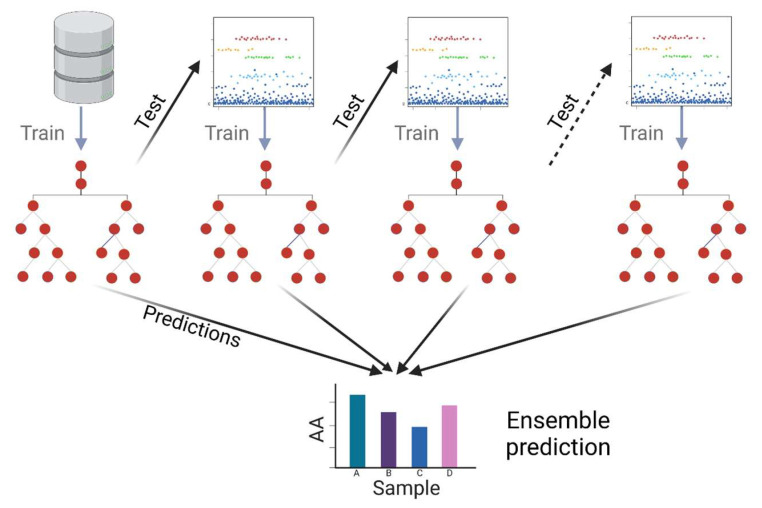
Schematic representation of the GBR tree.

The GB model yielded the best results out of the eight models initially tested, with a mean absolute error (MAE) of 9.027 and an R^2^ = 0.86. A gradient boosting regressor (GBR) is a machine learning algorithm used to solve regression problems, i.e., to predict a continuous numerical value as a function of a set of features. The central idea behind the GBR is to combine multiple weak regression models, usually shallow decision trees, into a more robust and accurate model. The process starts with a simple model that makes an initial prediction and then focuses on the residual errors of that initial prediction, successively fitting additional models to correct these errors. Each additional model is adjusted to focus on the instances incorrectly predicted in the previous step, and this iteration continues until a certain number of estimators is reached or satisfactory convergence is obtained. A schematic representation of this model can be observed in Figure 11. The result is a regressor combining the predictions of multiple weak models into a more accurate and robust prediction. The GBR stands out for its ability to handle nonlinear relationships in the data, its resistance to overfitting, and its ability to deal with noisy data. By fitting successive models based on residual errors, the model’s overall accuracy is improved, making it a valuable tool in solving regression problems in various fields, such as real estate price prediction, future income estimation, and many other applications.

The next step is to optimize the hyperparameters of the selected model. A technique called randomized search was used. Randomized search is a hyperparameter optimization technique in machine learning that helps identify the best combination of values for the hyperparameters of a model more efficiently than an exhaustive search. Instead of evaluating all possible combinations, randomized search randomly selects a set of hyperparameter combinations to evaluate, which saves on computational time. This approach is combined with cross-validation to measure model performance between each set of hyperparameters, and this is repeated several times with different random combinations. Ultimately, randomized search identifies the combination of hyperparameters that provides the best performance based on a predefined evaluation metric. This strategy allows for a more efficient and effective search for optimal hyperparameters, especially for problems where exhaustive exploration would be costly in terms of computational resources and time. The values for the best gradient boosting model were as follows: number of estimators: 1200; the minimum number of samples required to split an internal node: 5; the minimum number of samples required to reach a leaf: 5; maximum depth of the individual regression estimators: 4; loss function: Huber; and learning rate: 0.07. With these parameters, the model reaches a score of 0.95.

Finally, the feature importance analysis was applied (see Figure 10d). Feature importance is a measure used in machine learning to evaluate the relative contribution of each feature or variable in a prediction model. This metric helps to identify which features have a more significant impact on model predictions and which are less influential. It is generally calculated using random forests, gradient boosting models, or regression analysis. Features with greater importance often play a more significant role in the model’s ability to make accurate predictions, which can be helpful for feature selection, model interpretation, and decision making in real-world applications. The importance of each individual feature can be visualized in a bar chart showing its relative contribution to the model’s performance. In this type of representation, features with longer bars are the most important, significantly impacting the model’s predictions. Visualizing feature importance in this way helps data scientists and analysts understand which features are crucial in a model and, in turn, to make informed decisions about feature selection and fitting. For the case reported in this study, the variables with a higher importance were the DPPH method, nanoparticle concentration, TC, and the material’s charge.

## 4. Conclusions

In conclusion, ZnO-NPs doped with La^3+^ and Sm^3+^ were successfully synthesized using the polymerized solution method combined with ultrasonic treatment. Results regarding their antioxidant properties demonstrated that Z, ZL, and ZS are moderately potent antioxidants, as they inhibited the formation of DPPH radicals at IC_50_ values of 51.140 ± 14.985, 126.948 ± 5.811, and 99.707 ± 8.262 μg/mL, respectively. In contrast, ZLS can be considered a highly potent antioxidant due to its IC_50_ value of 3.898 ± 0.122 μg/mL. The evaluation of the nanomaterials’ anti-cancer activity revealed that treatment with Z, ZL, and ZLS can significantly decrease the viability of the MCF-7 cell line in a dose-dependent manner. Against the HT29 cell line, only treatment with Z can promote cell death. The calculated LC_50_ values against the MCF-7 cell line ranged from 161.418 ± 17.660 to 200.835 ± 57.778 μg/mL, whereas, against the HT29 cell line, the LC_50_ values ranged from 249.985 ± 93.527 to 504.917 ± 161.917 μg/mL. The cytotoxicity of the developed nanomaterials can be attributed to their capacity to decrease the levels of ROS within the two cancer cells, which was demonstrated by utilizing a fluorogenic probe, and suggest a possible mechanism by which doped ZnO-NPs can lead cancer cells to death. In the experiments with *A. salina* specimens, Z and ZS were found to accumulate inside their gut, but did not lead to their death. This effect was not observed during treatment with ZL and ZLS in the same assay. The results of this study expand the knowledge about the effect of doping elements in in vivo models and affirms the need to continue exploring the nanotoxicological properties of various nanomaterials.

## Figures and Tables

**Table 1:** Structural parameters of Z, ZL, ZLS, and ZS.

Material	*a* (Å)	*c* (Å)	Cell Volume (Å)^3^	Distortion
Z	3.243 (1)	5.199 (1)	4.735 (4)	1.019
ZL	3.240 (1)	5.200 (1)	4.726 (4)	1.017
ZLS	3.256 (1)	5.223 (1)	4.795 (4)	1.018
ZS	3.243 (1)	5.205 (1)	4.739 (4)	1.017

**Table 2:** LC_50_ values of Z, ZL, ZLS, and ZS against the MCF-7 and HT29 cell lines. Concentrations are expressed in μg/mL.

Material	MCF-7	HT29
Z	161.418 ± 17.660	249.985 ± 93.527
ZL	181.885 ± 9.455	504.917 ± 161.917
ZLS	174.982 ± 13.250	390.440 ± 116.376
ZS	200.835 ± 57.778	260.919 ± 24.677

## Data Availability

The data generated in this work can be consulted with the authors for correspondence of this work upon reasonable request.

## Abbreviations

RE	Rare earth
NPs	Nanoparticles
Zn^2+^	Zinc
La_2_O_3_	Lanthanum oxide
XRD	X-ray diffraction
ATR-FTIR	Attenuated total reflectance Fourier-transform infrared
FE-SEM	Field emission scanning electron microscopy
SBET	Specific surface area
UV–Vis	Ultraviolet–visible
PTFE	Polytetrafluoroethylene
DLS	Dynamic light scattering
DPPH	2,2-diphenyl-1-picrylhydrazyl
IC_50_	Half-maximal inhibitory concentration
LC_50_	Half-maximal lethal concentration
ATCC	American Type Culture Collection
DMEM	Dulbecco’s Modified Eagle’s Medium
FBS	Fetal bovine serum
PBS	Phosphate-buffered saline
MTT	Methyl-4,3-thiazolyltetrazolium
ELISA	Enzyme-linked immunosorbent assay
ROS	Reactive oxygen species
HGF-1	Human gingival fibroblasts
HUVECs	Human umbilical vein endothelial cells
BHK	Baby hamster kidney
Gs	Grain size
TC	Texture coefficient
EG	Band gap
Defects	Structural defects from EDS
DLS	Average particle size from DLS measurement
AA	Antioxidant activity
OHE	One-hot encoding
LR	Linear regression
RF	Random forest
ETs	Extremely random trees
DTs	Decision trees
MLP	Multi-layer perceptron
KNNs	K-nearest neighbors
GB or GBR	Gradient boosting
SVR	Support vector regressor
R2, R^2^	Correlation coefficient or determination coefficient.
MAE	Mean absolute error
MSE	Mean squared error
RMSE	Root mean squared error (standard error)
